# Advance of large-area achromatic flat lenses

**DOI:** 10.1038/s41377-023-01093-7

**Published:** 2023-03-01

**Authors:** Yubin Fan, Jin Yao, Din Ping Tsai

**Affiliations:** 1 Department of Electrical Engineering, City University of Hong Kong, Kowloon, Hong Kong 999077 China; 2 Centre for Biosystems, Neuroscience, and Nanotechnology, City University of Hong Kong, Kowloon, Hong Kong 999077 China; 3 The State Key Laboratory of Terahertz and Millimeter Waves, City University of Hong Kong, Kowloon, Hong Kong 999077 China

**Keywords:** Metamaterials, Imaging and sensing

## Abstract

A new framework of light coherence optimization is proposed to design non-ideal broadband achromatic lenses, enabling large-scale flat lenses’ implementation and high performance. The strategy paves the way for practical planar optical devices and full-color imaging systems.

## Abstract (web summary)

Non-ideal achromatic flat lenses for a large-scale diameter up to 10 mm were experimentally realized and reported recently, which demonstrated a broad working bandwidth from 400 to 1100 nm.

## Graphical abstract

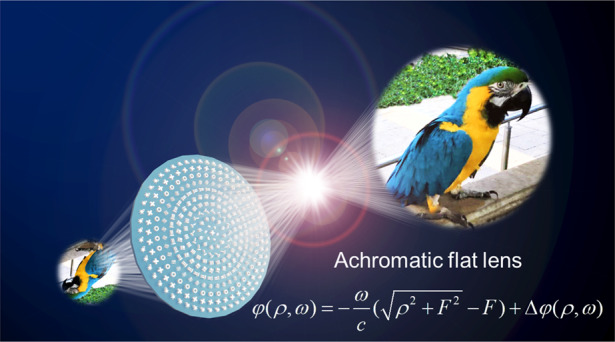

Metasurface shows its preeminent capabilities in wavefront manipulations and light-matter interactions. Various attempts have been carried out to realize applications based on metasurface. Above all these attempts from scientists and engineers, meta-lens is the most successful one. Meta-lens is a kind of flat lens made of subwavelength nanostructures. Starting from Fan et al. in 2009, metal nanostrips were involved in realizing one-dimensional focusing meta-lens^1^, offering guidelines for the lens design and showing how actual lens behavior deviates from simple theory. With the help of the geometric phase^2^, metal-antenna-supported plasmonic meta-lens can focus double charity polarities on both sides^3^, observing magnified and demagnified imaging at visible and near-infrared wavelengths. Since intrinsic losses of metals limit the development of plasmonic meta-lenses, all-dielectric materials with low extinction coefficients entered the researchers’ field of vision. Titanium dioxide (TiO_2_) meta-lens realized focusing and imaging at a single visible wavelength with an efficiency of over 50%^4^ in 2016. Multispectral chiral imaging meta-lens^5^ and immersion meta-lens^6^ based on TiO_2_ showed progress in the monochromatic meta-lens application. From classical to nonclassical scenarios, meta-lenses have also been applied in enhanced nonlinear generation^7^ and high dimensional quantum light source^8^. However, applications of meta-lens do not stop in a single wavelength. The development of an achromatic flat lens is highly demanding.

The attempt at achromatism was shown in a one-dimensional lens at a discrete wavelength. Results of three dispersed telecommunication wavelengths (1300, 1550, and 1800 nm) were focused at the same distance by coupled rectangular dielectric resonators^9,10^ in 2015. The coupling between meta-atoms provides a dense spectrum of optical modes to compensate for the dispersive phase difference. A two-dimensional lens is more feasible and widely used than a one-dimensional lens. By carefully designing the meta-molecule, two kinds of nanoantennas were integrated in one layer for two working wavelengths of 1550 and 915 nm with a numerical aperture (NA) of 0.46^11^, and the measured focusing efficiencies are 65 and 22%, respectively. Multilayer meta-lenses have a similar ability to focus red, green, and blue colors at the same position^12^. Each layer consists of meta-atoms made of dedicated plasmonic material and was designed to operate in a specific spectral band. The discrete achromatic correction is still far away from full color and wide-band imaging. Continuous achromatic meta-lens for full-color imaging is an important issue and highly demanded.

The bandwidth of achromatic meta-lenses experienced the development from narrow to wide. To begin with, by utilizing the optimization algorithm to select meta-atoms with the same phases while different dispersions, continuous achromatic meta-lens were demonstrated to have a 60 nm narrowband from 490 to 550 nm with an efficiency of 15%^13^. S. Wang et al.^14^ proposed the differential phase (DP) equation and combined both Pancharatnam-Berry (P-B) phase and resonance phase in the integrated-resonant units (IRUs). The phase compensation can be adjusted by controlling the coupling between plasmonic nanorods to achieve an achromatic meta-lens with bandwidth extended from 1200 to 1680 nm in reflective type with an efficiency of around 12%^14^. To meet the requirements of true color imaging, the development of achromatic meta-lens then entered a new era of continuous bandwidth achromatic meta-lens in the visible range. Aluminum-IRU was employed to construct broadband reflective meta-lens in the wavelength range of 420 to 650 nm^15^. The maximal focusing efficiency is around 26.31% with a NA = 0.124. With an average efficiency of 40% and a high transmission advantage, gallium nitride (GaN) achromatic meta-lens with nanopillars or nanoholes waveguide-resonances was developed for the visible working wavelength from 400 to 660 nm^16^ in 2018. Full-color imaging in transmission mode was successfully achieved in the continuous visible spectrum. A similar strategy of achromatism by controlling group delay dispersion was exploited to realize a TiO_2_ achromatic meta-lens from 470 to 670 nm with an efficiency of around 20% at 500 nm^17^. Achromatic meta-lens were then extended from circular polarization excitation to polarization insensitive. By creating libraries of meta-atoms with diverse phase dispersions, polarization-independent achromatic meta-lens were demonstrated to have a focusing efficiency up to 50% from 1200 to 1650 nm^18^. Y. Wang et al. further enhanced the average efficiency to 88.5% from 650 to 1000 nm and applied the TiO_2_ meta-lens in upconversion biological imaging with a high-resolution limit^19^. To facilitate the full-color light-field imaging applications, the meta-lens array contains an array of 60 × 60 GaN meta-lenses with a diameter of 21.65 μm was reported to reconstruct the depths of objects with a diffraction-limited resolution of 1.95 μm under white light illumination^20^. Based on the differentiated and rendering algorithm, the meta-lens array can be applied in one-dimensional to three-dimensional edge detection as well^21^. A multifunctional meta-lens array was further demonstrated for all light-level depth sensing. With the support of neural networks and deep learning, accurate depth mapping within the 21.0–50.5 cm working range can be achieved^22^. This meta-device can also work with a light source as an active optical device to project a structured light. Although various continuous broadband achromatic meta-lens and their versatile applications have been reported in the visible, the above works are based on the ideal lens design method. Significant phase compensation requirements may limit the size of meta-lens in experiments.

In 2022, Tao Li from Nanjing University and Guixin Li from Southern University of Science and Technology worked together to propose and experimentally realize non-ideal achromatic flat lenses for a large-scale diameter of up to 10 mm^23^. They solved the issues in two steps. First, they determined the fundamental limits on overall performance based on size, NA, and focal efficiency. Second, they propose an optimization framework for designing a feasible large-scale achromatic multi-level diffractive lens with optimal performance. Those large-scale achromatic flat lenses work in a super broad bandwidth from 400 to 1100 nm (Fig. 1).

**Fig. 1:**
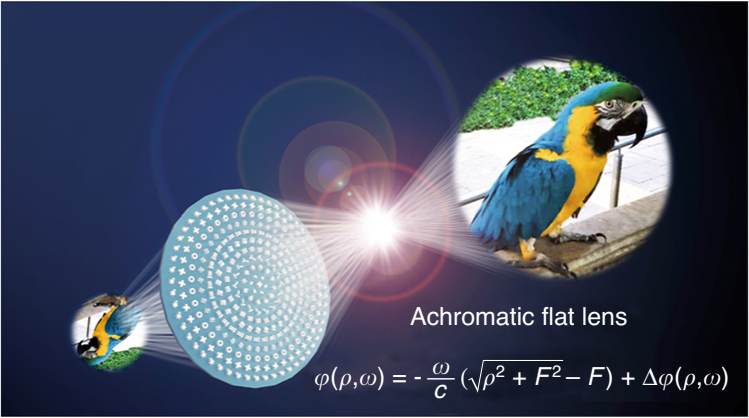
Operation principle of achromatic flat lenses. Schematic of large-area achromatic flat lenses

To design such large-scale achromatic flat lenses, they paid attention to the coherence in the frequency domain and involved mutual coherence function averaging in the frequency range of interest. The limits of NA, the diameter, and the height of the lens were derived and computed. Based on the equations, one can find the maximum diameter from a given maximum *n*(*ω*) over the whole spectrum, the required least thickness, and the highest NA. They also proposed a numerical approximation optimization framework for the engineering of mapping the corresponding phase with performance near the limit.

They used a positive polymer photoresist (AZ4562) layer and grayscale laser lithography technique for the fabrication. Unlike most of the previous works using all-dielectric high refractive index materials, their fabrication and material process allow fast and large-scale processing and inexpensive mass production. However, the grayscale laser lithography technique requires an aspect ratio under 2:1. Their proposed optimization framework has solved this issue by adding a smooth aspect ratio step. They calculated and showed the quasi-optimal height distribution of five samples for cases of S1–S5, respectively. The aspect ratio values of all the rings are less than 2:1, with their average efficiency around 68%, 42%, 31%, 18%, and 5%, respectively. Results are in good agreement with their calculation and simulation.

The realization of large-area flat lens applications^23^ successfully displays excellent potential and perspective of the achromatic flat lens. True color and continuous wide-band are primary for optical imaging. We trust that such flat meta-lenses can dramatically reduce the weight and the space of conventional bulky optical components. Achromatic flat lenses are thinner, compact, and able to manipulate the wavefront of light efficiently. Polymer materials in this work are less expensive in the fabrication process. After precise control of nanoimprinting technology, polymer materials will be more accessible to produce large-scale flat optical elements. Realizing large-scale flat lenses needs both fabrication and designing toolbox. Solving analytical formulas and numerical approximation optimization can achieve the design phase of achromatic flat lenses. The analytical solution gives more intuitive results and strictly required conditions, while the numerical optimization can provide optimal solutions under complex conditions but with less precision. These designing methods can improve imaging quality in many aspects besides flat lens designing, such as complementary metal–oxide–semiconductor (CMOS) pixel mapping and unitary design. Subsequent developments and applications are expected in various environments apart from on the ground, such as in the sky^24^ and under the water^25^. Artificial intelligence information can be integrated into the design and meta-optics applications^26^ as well. The perspective is not limited by intelligent designs and novel meta-devices but physics.
